# Blood viscosity as a continuous marker of cardio-metabolic risk burden: a large-scale cross-sectional study of 38,574 adults

**DOI:** 10.3389/fendo.2026.1861158

**Published:** 2026-07-31

**Authors:** Dayeon Lee, Byoungduck Han, Hongyup Ahn, Eunhye Cho, Minjung Kwon, Cheolyoung Park

**Affiliations:** 1 Division of Endocrinology and Metabolism, Department of Internal Medicine, Kangbuk Samsung Hospital, Sungkyunkwan University School of Medicine, Seoul, Republic of Korea; 2 Department of Family Medicine, Korea University Anam Hospital, Korea University College of Medicine, Seoul, Republic of Korea; 3 Department of Statistics, Dongguk University, Seoul, Republic of Korea; 4 Department of Laboratory Medicine, Kangbuk Samsung Hospital, Sungkyunkwan University School of Medicine, Seoul, Republic of Korea

**Keywords:** cardio-metabolic risk, hemorheology, metabolic burden, metabolic syndrome, whole blood viscosity

## Abstract

**Aims:**

Metabolic syndrome (MetS) is diagnosed using fixed thresholds that may not fully reflect the continuous accumulation of cardio-metabolic risk. We investigated whether whole blood viscosity (WBV) increases progressively with increasing MetS burden and explored the relative contribution of individual MetS components to hemorheologic alterations.

**Methods:**

We analyzed 38,574 adults (21,914 men and 16,660 women) who underwent comprehensive health examinations between January and April 2021. Whole blood viscosity was measured by cone-plate viscometry at shear rates of 300 s^-1^ (systolic blood viscosity [SBV]) and 5 s^-1^ (diastolic blood viscosity [DBV]). MetS was defined according to NCEP-ATP III criteria. Associations between WBV and MetS burden were evaluated using logistic regression, ANCOVA, regression tree analyses, ROC analyses, and sex-by-viscosity interaction testing.

**Results:**

WBV increased progressively with each additional MetS component in both sexes (p for trend <0.001). After full adjustment for hematocrit, age, smoking status, eGFR, white blood cell count, and AST/ALT, higher SBV was associated with MetS in both women (OR 1.45, 95% CI 1.32–1.58, per 1-SD increase) and men (OR 1.27, 95% CI 1.22–1.33, per 1-SD increase). Regression tree analyses identified triglycerides and waist circumference as metabolic factors associated with elevated WBV. Sex-stratified analyses demonstrated a stronger association between WBV and MetS in women than in men.

**Conclusions:**

WBV increased in parallel with accumulating cardio-metabolic risk burden, including among individuals who did not meet diagnostic criteria for MetS. WBV may serve as a complementary marker associated with metabolic risk burden, particularly among women and individuals with hypertriglyceridemia or central adiposity.

## Introduction

Cardiovascular disease (CVD) remains the leading cause of morbidity and mortality worldwide, and early identification of individuals at elevated cardio-metabolic risk is a central clinical priority (1). Metabolic syndrome (MetS)—a cluster of metabolic risk factors—including central obesity, hyperglycemia, hypertension, hypertriglyceridemia, and low high-density lipoprotein cholesterol— has been widely adopted as a clinical tool to identify individuals at increased risk of CVD and type 2 diabetes (2–4).

However, MetS relies on binary diagnostic thresholds that categorize individuals as either meeting or not meeting the criteria, potentially missing the continuum of rising risk that accumulates with each additional metabolic abnormality (5–8). Indeed, several researchers have noted that individuals with fewer than three criteria—and thus not classified as having MetS—may still harbor meaningful hemorheologic and vascular dysfunction (5, 6).

Whole blood viscosity (WBV) is a continuous measure of hemorheologic status, determined principally by plasma viscosity, hematocrit, and erythrocyte deformability (9, 10). Elevated WBV impairs microcirculatory flow, promotes endothelial dysfunction and oxidative stress, and accelerates atherogenesis (11, 12), with downstream contributions to adverse cardiovascular outcomes and mortality (13–17). Because WBV reflects the cumulative influence of metabolic, inflammatory, and rheological perturbations, it is a plausible integrative marker of cardio-metabolic burden—one that operates on a continuous scale rather than a binary threshold.

Prior studies have documented an association between WBV and MetS (9, 18–21); however, most were limited by small sample sizes, restriction to a single sex, reliance on calculated rather than directly measured viscosity, or analysis of MetS as a binary outcome without examining the progressive association with individual components. Critically, whether WBV differs across progressive levels of metabolic risk burden, including subthreshold MetS states —as early as the first or second component—has not been adequately characterized in large-scale cohorts.

Therefore, the present study aimed to determine whether directly measured BV increases as a continuous function of accumulating cardio-metabolic risk burden—as operationalized by the individual MetS components (hyperglycemia, hypertension, dyslipidemia, and clinical obesity defined by abdominal circumference)—in a large Korean cohort. We further sought to identify sex-specific patterns in this relationship and to rank the relative contribution of individual components to hemorheologic burden using regression tree analysis.

## Research design and methods

### Study population

This cross-sectional study was conducted using data from adults who underwent comprehensive health examinations at the Health Screening Center of Kangbuk Samsung Hospital, Seoul, Republic of Korea. Data were collected from January 4, 2021 to April 30, 2021. Eligible participants were adults aged 18 years or older who completed both WBV measurement and full metabolic panel testing as part of their routine health examination. Because the analyses were based on complete health-screening datasets routinely collected within the examination program, no participants were excluded due to missing data for MetS components or WBV measurements. Of the initial 39,115 individuals screened, participants currently using anticoagulant or antiplatelet medications—including warfarin, aspirin, and other antithrombotic agents—were excluded (n = 541), as these medications may directly influence blood viscosity and hemorheologic measurements independent of metabolic status. The final study population consisted of 38,574 individuals (**Supplementary Figure 1**). This study was approved by the Institutional Review Board of Kangbuk Samsung Hospital (IRB No. 2021-05-010).

### Clinical and biochemical measurements

Anthropometric assessments included measurements of height, body weight, and body mass index (BMI; weight [kg] divided by height [m^2^]). Blood pressure was measured on the right arm after at least five minutes of seated rest, and systolic (SBP) and diastolic (DBP) blood pressure values were recorded. Smoking status was assessed by self-reported questionnaire; participants smoking at least one cigarette per day in the preceding 12 months were classified as current smokers.

Serum total cholesterol, triglycerides, HDL-cholesterol, LDL-cholesterol, AST, ALT, GGT, glucose, creatinine, and uric acid were measured on the Cobas c702 analyzer (Roche Diagnostics, Tokyo, Japan). HbA1c was measured by turbidimetric inhibition immunoassay on the Cobas c513 analyzer (Roche Diagnostics). eGFR was calculated using the 2009 CKD-EPI creatinine equation. Complete blood count including WBC, RBC, hemoglobin, and hematocrit was measured using the Sysmex XE-2100D Automated Hematology Analyzer (Sysmex Corporation, Kobe, Japan).

Whole blood viscosity (WBV) was measured directly using the cone-plate method with the ZL6000P Blood Rheology Analyzer (Zonci Technology, Beijing, China) as part of a routine health examination program under standardized laboratory procedures. Venous blood samples collected in K2-EDTA tubes were processed according to a standardized laboratory protocol. Measurements were performed at shear rates of 300 s^-1^ and 5 s^-1^, which were defined as systolic blood viscosity (SBV) and diastolic blood viscosity (DBV), respectively. Daily quality-control procedures demonstrated between-day coefficients of variation of 2.90% for SBV and 3.24% for DBV.

Given the large study population, additional details regarding laboratory workflow, measurement throughput, analyzer availability, laboratory operating schedule, sample handling, temperature control, calibration, quality assurance and reference interval establishment are provided in the **Supplementary Methods** (22).

### Definition of metabolic syndrome

MetS was defined using modified National Cholesterol Education Program Adult Treatment Panel III (NCEP-ATP III) criteria, requiring three or more of the following five components: waist circumference >90 cm in men or >85 cm in women; fasting triglycerides ≥150 mg/dL; HDL-cholesterol <40 mg/dL in men or <50 mg/dL in women; SBP ≥130 mmHg and/or DBP ≥85 mmHg, or current antihypertensive medication use; and fasting glucose ≥100 mg/dL or use of hypoglycemic agents (23). Only the waist circumference cut-off was modified from the original NCEP-ATP III definition (>102/88 cm), using Korea-specific thresholds recommended by the Korean Society for the Study of Obesity, as Koreans develop metabolic risk at lower levels of adiposity than Western populations (24). In addition to the binary MetS classification, the number of fulfilled MetS components (0–5) was used as a continuous exposure variable to characterize the progressive association with WBV.

### Statistical analysis

Continuous variables are presented as mean ± standard deviation and categorical variables as frequency (percentage). Sex differences in baseline characteristics were assessed using independent-samples t-tests and chi-square tests, as appropriate. The primary analysis compared WBV across increasing numbers of MetS components (0–5) using one-way ANOVA with *post-hoc* pairwise comparisons. Analysis of covariance (ANCOVA) was used to compare adjusted mean SBV and DBV between MetS(+) and MetS(−) groups, with hematocrit and sex as covariates.

Logistic regression was used to estimate odds ratios (ORs) and 95% confidence intervals (CIs) for the association between WBV and prevalent MetS, stratified by sex. Three sequential models were constructed: Model 1 (unadjusted), Model 2 (adjusted for hematocrit), and Model 3 (fully adjusted for hematocrit, age, smoking status, estimated glomerular filtration rate [eGFR], white blood cell count, and AST/ALT). SBV and DBV were modeled as continuous variables, and odds ratios were calculated per 1-SD increase in blood viscosity. The optimal number of MetS components to define high-risk subgroups was determined using the Akaike Information Criterion (AIC). As sensitivity analyses, a weighted MetS score was derived from a crude logistic regression model using the cardiovascular disease (CVD) variable available in the health-screening dataset as the outcome, and its association with blood viscosity was evaluated using crude linear regression. Multivariable linear regression analyses including all five MetS components simultaneously were additionally performed to estimate their independent contributions to SBV and DBV. To identify the relative contribution of individual MetS components to WBV, regression tree analysis was performed on the full study population using recursive partitioning (rpart package, R). Sex was pre-specified as the first branching variable, given its established influence on baseline blood viscosity. Binary indicators of individual MetS components — triglyceride criterion (tgC), waist circumference criterion (wcC), and fasting glucose criterion (glucC) — were entered as candidate splitting variables. Tree pruning was performed using a complexity parameter of cp = 0.001, resulting in six terminal nodes for SBV and five terminal nodes for DBV (**Supplementary Figure 2**). Given known collinearity among MetS components (e.g., between waist circumference and triglycerides), the regression tree findings were interpreted as exploratory rather than definitive evidence of hemorheologic dominance. All analyses were performed using R version 4.2.3 (http://www.r-project.org) with a two-sided significance level of 0.05.

## Results

### Baseline characteristics of subjects according to sex and MetS

Baseline characteristics stratified by sex and MetS status are summarized in **Table 1**; overall baseline characteristics of the total study population by sex are provided in **Supplementary Table 1**. The prevalence of MetS was higher in men than in women (16.0% vs. 5.4%). Participants with MetS had higher age, hematocrit, SBV, and DBV compared with those without MetS across both sexes. Among those with MetS, men had significantly higher waist circumference and triglyceride levels than women (95.5 ± 9.7 vs. 90.6 ± 8.9 cm; 222.5 ± 117.8 vs. 184.5 ± 107.4 mg/dL), whereas fasting glucose and HbA1c were higher in women with MetS (111.4 ± 26.4 mg/dL and 6.03 ± 1.03%) than in men with MetS (108.6 ± 19.9 mg/dL and 5.82 ± 0.77%).

**Table 1:** Clinical and biochemical characteristics of subjects according to sex and MetS.

Variable	Men	Men	Women	Women	P value for sex	P value for MetS
	MetS-	MetS+	MetS-	MetS+		
n	18,295	3,619	15,755	905		
Age, years	40.44 ± 8.45	43.78 ± 8.41	40.16 ± 8.70	47.84 ± 12.44	p<0.001	p<0.001
BMI, kg/m^2^	24.49 ± 2.77	27.90 ± 3.04	21.73 ± 3.02	27.57 ± 4.16	p<0.001	p<0.001
Waist circumference, cm	85.83 ± 7.54	95.49 ± 7.11	74.93 ± 7.94	90.62 ± 8.86	p<0.001	p<0.001
Systolic BP, mmHg	111.16 ± 9.59	118.94 ± 11.01	103.71 ± 10.79	119.75 ± 14.50	p<0.001	p<0.001
Diastolic BP, mmHg	70.49 ± 7.34	76.36 ± 8.29	66.13 ± 7.41	74.56 ± 9.61	p<0.001	p<0.001
Total cholesterol, mg/dL	196.09 ± 33.10	199.15 ± 38.27	191.75 ± 32.76	199.16 ± 39.98	p<0.001	p<0.001
HDL-c, mg/dL	56.42 ± 12.49	44.91 ± 10.34	71.13 ± 15.39	50.16 ± 11.81	p<0.001	p<0.001
LDL-c, mg/dL	132.83 ± 31.53	132.50 ± 34.92	119.44 ± 30.58	133.03 ± 37.63	p<0.001	p<0.001
Triglyceride, mg/dL	116.43 ± 63.85	222.52 ± 117.76	83.08 ± 43.06	184.51 ± 107.42	p<0.001	p<0.001
Fasting Glucose, mg/dL	95.48 ± 10.13	108.56 ± 19.86	92.09 ± 9.73	111.41 ± 26.36	p<0.001	p<0.001
HbA1c, %	5.43 ± 0.39	5.82 ± 0.77	5.32 ± 0.34	6.03 ± 1.03	p<0.001	p<0.001
eGFR, mL/min/1.73m^2^	95.72 ± 14.53	95.74 ± 17.81	104.75 ± 18.81	101.61 ± 20.45	p<0.001	p<0.001
Uric acid, mg/dL	6.16 ± 1.21	6.59 ± 1.36	4.31 ± 0.92	5.05 ± 1.17	p<0.001	p<0.001
AST, IU/L	24.66 ± 18.02	30.96 ± 16.00	19.65 ± 15.46	26.33 ± 16.99	p<0.001	p<0.001
ALT, IU/L	29.20 ± 21.33	45.26 ± 30.64	15.61 ± 14.46	30.24 ± 27.74	p<0.001	p<0.001
GGT, IU/L	34.36 ± 33.48	57.92 ± 50.77	17.02 ± 16.20	33.04 ± 35.85	p<0.001	p<0.001
Protein, g/dL	7.46 ± 0.38	7.49 ± 0.37	7.40 ± 0.41	7.51 ± 0.40	p<0.001	p<0.001
Albumin, g/dL	4.88 ± 0.25	4.86 ± 0.24	4.73 ± 0.26	4.69 ± 0.25	p<0.001	p<0.001
WBC, x10^3^/uL	5.68 ± 1.40	6.31 ± 1.50	5.56 ± 1.52	6.61 ± 1.65	p<0.001	p<0.001
RBC, x10^6^/uL	5.07 ± 0.32	5.13 ± 0.33	4.46 ± 0.30	4.63 ± 0.35	p<0.001	p<0.001
Hb, g/dL	15.53 ± 0.88	15.70 ± 0.94	13.39 ± 1.06	13.75 ± 1.13	p<0.001	p<0.001
Hct, %	45.42 ± 2.38	45.68 ± 2.50	40.07 ± 2.77	41.03 ± 2.96	p<0.001	p<0.001
SBV, mPa·s	4.05 ± 0.44	4.19 ± 0.51	3.52 ± 0.36	3.75 ± 0.51	p<0.001	p<0.001
DBV, mPa·s	10.37 ± 1.79	10.60 ± 1.43	8.97 ± 1.14	9.43 ± 1.49	p<0.001	p<0.001
Smoking, n	2, 537 (13.87%)	734 (20.28%)	196 (1.24%)	15 (1.66%)	p<0.001	p<0.001

Data are presented as mean ± standard deviation (SD) or number (%). Continuous variables were compared using independent-samples *t*-tests, and categorical variables were compared using the χ^2^ test. BMI, body mass index; SBP, systolic blood pressure; DBP, diastolic blood pressure; HDL-c, high-density lipoprotein cholesterol; LDL-c, low-density lipoprotein cholesterol; AST, aspartate aminotransferase; ALT, alanine aminotransferase; GGT, gamma-glutamyl transferase; eGFR, estimated glomerular filtration rates; WBC, white blood cell count; RBC, red blood cell count; Hb, Hemoglobin; Hct, hematocrit; SBV, systolic blood viscosity; DBV, diastolic blood viscosity; MetS, metabolic syndrome.

### Progressive increase in blood viscosity across the cardio-metabolic risk continuum

**Figure 1:**
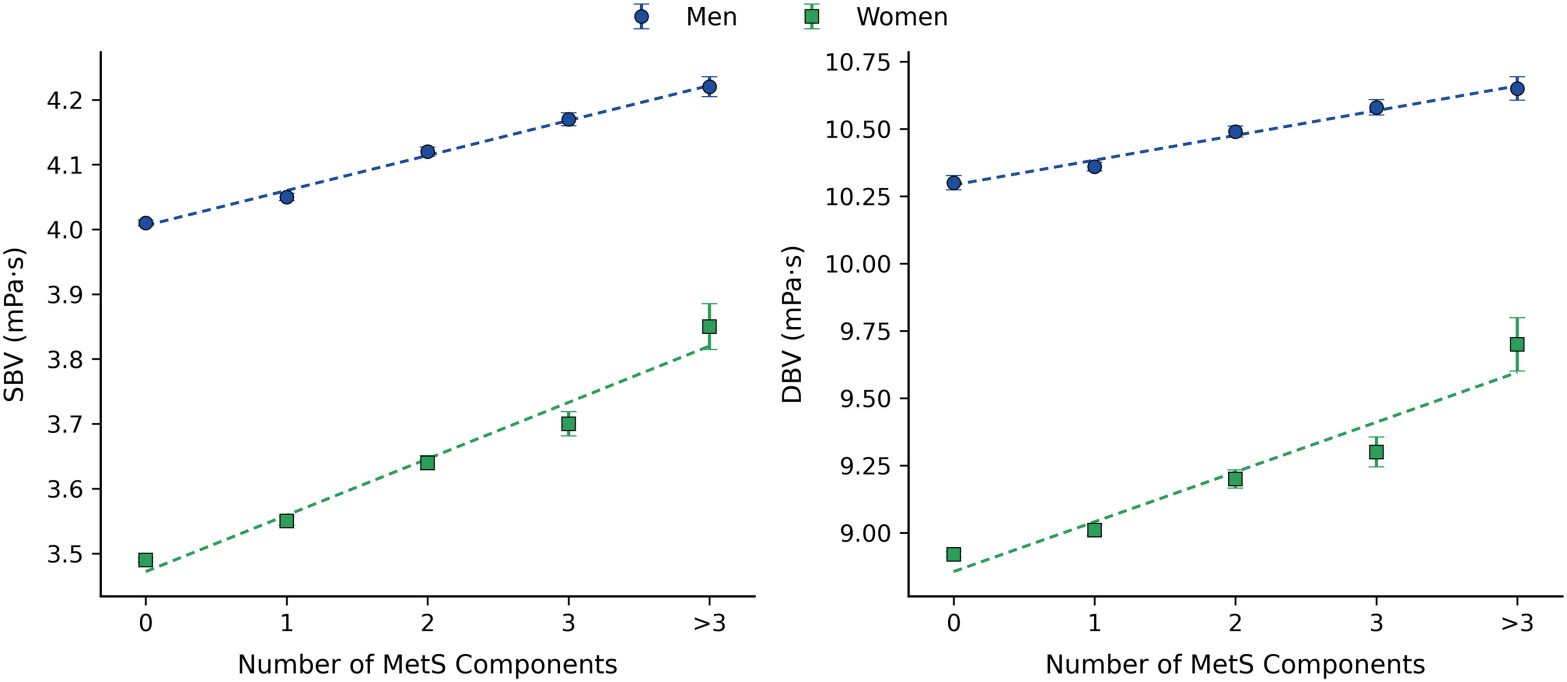
Blood viscosity according to number of metabolic syndrome components (total). Points represent mean SBV and DBV with 95% confidence intervals (CIs) for men (blue) and women (green). Dashed lines indicate fitted linear trends across increasing numbers of MetS components. Group comparisons were performed using one-way ANOVA with *post-hoc* pairwise comparisons. SBV, systolic blood viscosity; DBV, diastolic blood viscosity; CI, confidence interval.

Blood viscosity increased progressively with the number of MetS components in both sexes (**Figure 1**; **Table 2**). Among men, SBV rose from 4.01 ± 0.40 mPa·s in those with no components to 4.22 ± 0.51 mPa·s in those with ≥3 components; DBV rose from 10.30 ± 2.29 to 10.65 ± 1.44 mPa·s. Among women, SBV increased from 3.49 ± 0.34 to 3.85 ± 0.59 mPa·s and DBV from 8.92 ± 1.10 to 9.70 ± 1.66 mPa·s. Importantly, BV was significantly elevated even in individuals with only a single MetS component compared with those with no MetS components (p<0.001 for trend in both sexes). The DBV/SBV ratio, reflecting shear-thinning behavior, showed only statistically significant but numerically modest variation across increasing MetS component burden in both sexes (**Table 2**).

**Table 2:** Blood viscosity according to number of metabolic syndrome components.

Sex	Variable	0 MetS components	1 MetS component	2 MetS components	3 MetS components	>3 MetS components	P value
Men	n	7, 547	6, 312	4, 436	2, 515	1, 104	
	SBV, mPa·s	4.01 ± 0.40	4.05 ± 0.44	4.12 ± 0.48	4.17 ± 0.50	4.22 ± 0.51	<0.001
	DBV, mPa·s	10.30 ± 2.29	10.36 ± 1.28	10.49 ± 1.37	10.58 ± 1.43	10.65 ± 1.44	<0.001
	DBV/SBV ratio	2.57 ± 0.68	2.56 ± 0.17	2.55 ± 0.16	2.53 ± 0.15	2.52 ± 0.15	<0.001
Women	n	10, 842	3, 496	1, 417	626	279	
	SBV, mPa·s	3.49 ± 0.34	3.55 ± 0.38	3.64 ± 0.42	3.70 ± 0.47	3.85 ± 0.59	<0.001
	DBV, mPa·s	8.92 ± 1.10	9.01 ± 1.18	9.20 ± 1.27	9.30 ± 1.39	9.70 ± 1.66	<0.001
	DBV/SBV ratio	2.55 ± 0.17	2.54 ± 0.17	2.53 ± 0.17	2.51 ± 0.17	2.52 ± 0.17	<0.001

Data are presented as mean ± standard deviation (SD). P values were calculated using one-way ANOVA with *post-hoc* pairwise comparisons. MetS, metabolic syndrome; SBV, systolic blood viscosity; DBV, diastolic blood viscosity.

### Validation of the progressive association using a weighted MetS component model

To determine whether the observed graded association between metabolic burden and blood viscosity was robust to differential weighting of individual MetS components, a weighted MetS score was constructed using regression coefficients derived from a crude logistic regression model using the cardiovascular disease (CVD) variable available in the health-screening dataset as the outcome (**Supplementary Table 2**, Part A). Despite differential weighting of the individual MetS components, the weighted MetS score remained significantly associated with both SBV and DBV (**Supplementary Table 2**, Part B), supporting the robustness of the primary findings.

To further evaluate the independent contribution of each MetS component, multivariable linear regression analyses including all five MetS components simultaneously were performed. All five MetS components were independently associated with both SBV and DBV, with the HDL-cholesterol criterion demonstrating an inverse association (all p < 0.001; **Supplementary Table 3**).

### Blood viscosity in relation to cardio-metabolic risk states including metabolic syndrome

Consistent with the progressive increase observed across MetS component burden, blood viscosity was also significantly associated with prevalent MetS. In unadjusted logistic regression analyses (Model 1), higher SBV and DBV were significantly associated with prevalent MetS in both sexes, with a substantially stronger association in women (SBV: OR 1.743, 95% CI 1.630–1.864; DBV: OR 1.615, 95% CI 1.487–1.753) than in men (SBV: OR 1.319, 95% CI 1.274–1.366; DBV: OR 1.186, 95% CI 1.136–1.238) (**Table 3**). After adjustment for hematocrit (Model 2), these associations remained statistically significant in all groups, although the magnitude of association changed differentially by sex and viscosity parameter. Hematocrit adjustment resulted in attenuation of the association in women (SBV: OR 1.743 → 1.643; DBV: OR 1.615 → 1.402) but had minimal impact in men (SBV: OR 1.319 → 1.347; DBV: OR 1.186 → 1.151), suggesting that hematocrit may partially mediate the relationship between MetS and blood viscosity, particularly in women. After further adjustment for age, smoking status, eGFR, white blood cell count, and AST/ALT (Model 3), the associations remained significant but were further attenuated (Men SBV: OR 1.271, 95% CI 1.217–1.328; Men DBV: OR 1.073, 95% CI 1.029–1.137; Women SBV: OR 1.446, 95% CI 1.321–1.581; Women DBV: OR 1.283, 95% CI 1.153–1.426), with the association in women remaining approximately 1.1- to 1.2-fold stronger than in men (**Table 3**). Formal sex-by-viscosity interaction analyses demonstrated that these associations were significantly stronger in women than in men across all adjustment levels (SBV×sex: Model 1 OR 1.321, 95% CI 1.255–1.425; Model 2 OR 1.488, 95% CI 1.248–1.774; Model 3 OR 1.166, 95% CI 1.058–1.284; DBV×sex: unadjusted OR 1.362, 95% CI 1.241–1.494; Hct-adjusted OR 1.126, 95% CI 1.053–1.202; fully adjusted OR 1.211, 95% CI 1.077–1.357; all p<0.001) (**Supplementary Table 4**). Consistent findings were observed in complementary ANCOVA analyses adjusted for hematocrit and sex, which confirmed that MetS was independently associated with elevated WBV (**Supplementary Table 5**). Notably, the sex difference in SBV observed in the MetS(−) group was attenuated in the MetS(+) group.

**Table 3:** Blood viscosity in relation to cardio-metabolic risk states including metabolic syndrome.

Group	Variable	OR (95% CI) model 1 (unadjusted)	OR (95% CI) model 2 (Hct adjusted)	OR (95% CI) model 3 (fully adjusted)	P value
Men (n = 21,914)	SBV	1.319 (1.274-1.366)	1.347 (1.294-1.402)	1.271 (1.217-1.328)	<0.001
	DBV	1.186 (1.136-1.238)	1.151 (1.094-1.210)	1.073 (1.029-1.137)	<0.001
Women (n = 16,660)	SBV	1.743 (1.630-1.864)	1.643 (1.520-1.777)	1.446 (1.321-1.581)	<0.001
	DBV	1.615 (1.487-1.753)	1.402 (1.270-1.545)	1.283 (1.153-1.426)	<0.001

Odds ratios (ORs) were estimated using sex-stratified logistic regression models. Model 1: unadjusted; Model 2: adjusted for hematocrit; Model 3: adjusted for hematocrit, age, smoking status, estimated glomerular filtration rate (eGFR), white blood cell count (WBC), AST, and ALT. ORs are presented per 1-SD increase in SBV or DBV. OR, odds ratio; CI, confidence interval; SBV, systolic blood viscosity; DBV, diastolic blood viscosity; Hct, hematocrit; eGFR, estimated glomerular filtration rate; WBC, white blood cell count.

### Key metabolic syndrome components affecting blood viscosity

Having established a graded association between blood viscosity and metabolic burden, regression tree analyses were performed to identify which individual metabolic abnormalities were most consistently associated with this relationship. The regression tree analysis of the full study population identified sex as the primary branching variable for both SBV and DBV (**Supplementary Figure 2**). Among women, triglyceride criterion (tgC) emerged as the primary metabolic determinant of SBV; those meeting the triglyceride criterion had markedly higher SBV (3.673 ± 0.012 mPa·s) compared with those without triglyceride abnormality (3.515 ± 0.003 mPa·s), with fasting glucose criterion (glucC) providing further differentiation in the latter group. Among men, triglycerides similarly emerged as the second-level splitting variable, with waist circumference criterion (wcC) providing additional stratification among those without triglyceride abnormality (SBV 4.009 ± 0.004 vs. 4.097 ± 0.007 mPa·s for wcC=0 vs. 1, respectively); the highest SBV was observed in men meeting the triglyceride criterion (4.166 ± 0.006 mPa·s, n=6, 609).

For DBV, the splitting hierarchy diverged by sex. Among women, waist circumference was the primary metabolic determinant (DBV 8.954 ± 0.010 vs. 9.215 ± 0.027 mPa·s for wcC=0 vs. 1). Among men, triglycerides again emerged as the primary splitting variable, with the highest DBV observed in those meeting the triglyceride criterion (10.581 ± 0.017 mPa·s, n=6, 609); waist circumference provided secondary differentiation among men without triglyceride abnormality (DBV 10.273 ± 0.019 vs. 10.469 ± 0.021 mPa·s for wcC=0 vs. 1).

### Diagnostic performance of WBV for metabolic syndrome

Because WBV showed consistent associations across the continuum of metabolic burden and with prevalent MetS, ROC analyses were subsequently performed to evaluate its discriminatory performance for prevalent MetS. In the total population, SBV demonstrated moderate discriminatory performance (AUC 0.687, 95% CI 0.680–0.695, sensitivity 73.5%, specificity 54.6%). DBV showed comparatively lower discrimination (AUC 0.662, 95% CI 0.654–0.670; sensitivity 82.4%, specificity 44.5%). Sex-stratified analyses demonstrated higher discriminatory performance of SBV in women (AUC 0.659, 95% CI 0.640–0.677) than in men (AUC 0.594, 95% CI 0.584–0.604) (**Supplementary Table 6**).

Comparative reclassification analyses demonstrated that SBV provided significantly better discrimination than DBV (NRI −0.454, 95% CI −0.528 to −0.370, p<0.001; IDI −0.013, 95% CI −0.017 to −0.010, p<0.001). Furthermore, SBV showed incremental discriminatory value over the TG/HDL-C ratio (NRI 1.210, 95% CI 1.176–1.238, p<0.001; IDI 0.258, 95% CI 0.242–0.273, p<0.001), suggesting that WBV provides additive information beyond established lipid-based surrogates (**Supplementary Table 7**).

## Discussion

In this large-scale cross-sectional analysis of 38, 574 Korean adults, we demonstrate that BV rises progressively with each additional MetS component, including among individuals who did not meet the diagnostic criteria for MetS. Sensitivity analyses using alternative weighting approaches and multivariable models yielded consistent findings, supporting the robustness of the observed graded association. Together, these findings suggest that blood viscosity is closely associated with increasing metabolic burden across the cardio-metabolic risk continuum.

This continuum-based perspective has important clinical implications. Current MetS criteria require at least three of five components, and individuals with one or two components are typically not flagged for intensified intervention. However, in the present study, higher blood viscosity was consistently observed even among individuals with only one or two MetS components, indicating that hemorheologic alterations are already detectable in individuals who do not meet the full diagnostic threshold for MetS. Rather than simply differentiating individuals according to the binary presence or absence of MetS, WBV appears to provide complementary hemorheologic information across a spectrum of metabolic dysfunction (11, 12). Because the individual components of MetS—hyperglycemia, hypertension, dyslipidemia, and central obesity—represent the biologically active drivers of cardio-metabolic risk (25), WBV may therefore serve as an integrative hemorheologic measure associated with overall cardio-metabolic impairment.

To further explore the potential clinical utility of WBV, we evaluated its diagnostic performance for prevalent MetS as a secondary analysis. SBV demonstrated moderate discriminatory performance, whereas DBV consistently showed weaker discrimination. Reclassification analyses likewise suggested that SBV provided modest incremental discriminatory information compared with DBV and the TG/HDL-C ratio (26). However, because TG and HDL-C are themselves components of the MetS definition, these comparisons should be interpreted cautiously and regarded as exploratory rather than as evidence that WBV outperforms established metabolic indices. Taken together, these findings suggest that WBV may provide complementary hemorheologic information beyond conventional metabolic assessment, rather than serving as a standalone screening tool for MetS. Whether incorporation of WBV into clinical risk assessment provides incremental prognostic information beyond established metabolic markers remains to be determined in prospective studies.

The differential performance of SBV and DBV provides additional insight into the hemorheologic changes accompanying metabolic dysfunction. Although low-shear viscosity is theoretically more relevant to microcirculatory impairment (27), the associations observed for DBV were consistently weaker than those for SBV. This difference may reflect the greater biological variability of DBV under low-flow conditions, whereas SBV is thought to more consistently capture systemic rheologic alterations associated with metabolic dysfunction (12, 28, 29) Consistent with this interpretation, the minimal variation in the DBV/SBV ratio, which reflects shear-thinning behavior, suggests that both low- and high-shear viscosity increased proportionally rather than indicating preferential amplification of low-shear rheologic behavior. However, these mechanistic interpretations remain speculative and require confirmation in dedicated mechanistic studies.

Because hematocrit is a major determinant of blood viscosity and may also be influenced by metabolic dysfunction, we examined the impact of sequential covariate adjustment on these associations. Hematocrit adjustment attenuated the association primarily in women (SBV: OR 1.743→1.643; DBV: OR 1.615→1.402), whereas associations in men were minimally affected (SBV: OR 1.319→1.347). These findings are consistent with the possibility that hematocrit partially mediates, rather than simply confounds, the association between metabolic burden and blood viscosity, particularly in women (30). Further adjustment for age, smoking status, renal function, inflammatory status, and liver enzymes resulted in additional attenuation in both sexes (fully adjusted SBV: men OR 1.271, women OR 1.446; fully adjusted DBV: men OR 1.073, women OR 1.283), yet the associations remained statistically significant and the relative sex difference persisted. Overall, these findings indicate that the association between blood viscosity and metabolic abnormalities remained significant after sequential adjustment, supporting the robustness of the observed association across multiple clinically relevant covariates.

This association also differed notably by sex. Although baseline BV was higher in men—consistent with higher hematocrit levels (31)—the association between WBV and MetS was stronger in women. This finding may partly reflect the lower prevalence of MetS among women (5.4% vs. 16.0%), such that women who met the diagnostic criteria may represent a subgroup with relatively greater metabolic and hemorheologic impairment. Women also have lower baseline BV because of physiological and hormonal differences, including estrogen-mediated vascular protection (32), which may enhance the relative responsiveness of WBV to metabolic abnormalities (33, 34). However, these interpretations remain hypothesis-generating because detailed hormonal information, including menopausal status, menstrual status, oral contraceptive use, and hormone replacement therapy, was unavailable.

To further characterize the metabolic profile associated with higher blood viscosity, we performed regression tree analyses to identify the metabolic abnormalities most strongly associated with elevated WBV. Triglycerides and waist circumference consistently emerged as the principal determinants of blood viscosity. These findings are biologically plausible, as hypertriglyceridemia increases plasma viscosity and impairs erythrocyte deformability (35, 36), whereas central adiposity promotes insulin resistance and hemorheologic disturbances (37). Although systolic blood pressure may also contribute (38), triglycerides and waist circumference remained the dominant variables in our models. However, because of the substantial collinearity among MetS components, the splitting hierarchy should be interpreted as exploratory rather than definitive evidence of biological dominance.

This study has several strengths. First, WBV was directly measured using a standardized cone-plate viscometer rather than estimated computationally, providing a more direct assessment of hemorheologic properties. Second, rather than evaluating WBV solely in relation to the binary presence or absence of MetS, we examined its association across the full spectrum of metabolic abnormalities defined by the accumulation of MetS components. Third, the large sample size, comprehensive sex-stratified analyses, and use of a large occupational cohort with complete WBV and metabolic data minimized missing-data bias. Finally, regression tree analyses provided a complementary, data-driven approach for exploring the relative contribution of individual metabolic abnormalities to blood viscosity, thereby generating hypotheses regarding the metabolic profile associated with elevated WBV.

We also acknowledge several limitations. The cross-sectional design precludes causal inference and does not permit assessment of incident cardiovascular outcomes. The study was conducted at a single occupational health-screening center, which may have introduced a healthy-worker effect and self-selection bias, thereby limiting the generalizability of our findings. Furthermore, because body composition, metabolic phenotypes, and BMI distributions differ across ethnic groups, findings from this Korean cohort may not be directly generalizable to Western, South Asian, or other multi-ethnic populations. Information regarding lipid-lowering, antihypertensive, hypoglycemic, oral contraceptive, and hormone replacement therapy use was unavailable, precluding medication-specific sensitivity analyses. Detailed hormonal information, including menopausal status, was also unavailable. Finally, although the regression tree models were internally cross-validated, the findings should be regarded as exploratory because of potential collinearity among MetS components and the absence of external validation. Future prospective studies in community-based and multi-ethnic populations incorporating detailed medication histories, hormonal status, and longitudinal cardiovascular outcomes are warranted to validate and extend these findings.

In conclusion, WBV may serve as a continuous marker of cardio-metabolic risk burden that rises progressively with accumulating metabolic abnormalities, across the full spectrum of metabolic risk burden. It offers a complementary, non-invasive hemorheologic dimension to conventional metabolic risk assessment—particularly in women and in individuals with hypertriglyceridemia or central adiposity. Future prospective studies should determine whether WBV-guided risk stratification improves risk prediction of long-term cardio-metabolic outcomes.

## Data Availability

The data analyzed in this study is subject to the following licenses/restrictions: The data are not publicly available due to privacy and ethical restrictions involving patient information. Requests to access these datasets should be directed to CP, cydoctor@skku.edu.
