# Supplementary material for: Blood viscosity as a continuous marker of cardio-metabolic risk burden: a large-scale cross-sectional study of 38,574 adults
Source: Front Endocrinol (Lausanne). 2026 Jul 31;17:1861158. doi: 10.3389/fendo.2026.1861158 (PMC13472914; doi:10.3389/fendo.2026.1861158)
Supplement: Supplementary file 1 [file SupplementaryFile1.docx]

**Supplementary Materials**

**Contents**

Supplementary Figure 1. **Flow diagram of study participant selection**	2

Supplementary Methods. **Whole blood viscosity measurement**	3

Supplementary Table 1. **Baseline characteristics of the overall study population**	5

Supplementary Table 2. **Sensitivity analyses using weighted MetS component models of CVD**	6

Supplementary Table 3. **Independent contributions of individual metabolic syndrome components to systolic and diastolic blood viscosity**	7

Supplementary Table 4. **Sex-by-viscosity interaction analyses for metabolic syndrome**	8

Supplementary Table 5. **Difference in blood viscosity between participants with and without metabolic syndrome**	9

Supplementary Table 6. **Diagnostic Performance of WBV for Metabolic Syndrome**	10

Supplementary Table 7. **Comparative reclassification analyses**	11

Supplementary Figure 2. **Regression tree analysis identifying key metabolic determinants of blood viscosity**	12

**Supplementary Figure 1. Flow diagram of study participant selection.**

## Supplementary Methods

## Whole blood viscosity measurement

Whole blood viscosity (WBV) was measured using the ZL6000P Blood Rheology Analyzer (Zonci Technology, Beijing, China) based on the cone-plate viscometry principle.

Venous blood samples were collected into vacuum tubes containing K2-ethylenediaminetetraacetic acid (EDTA) anticoagulant. Samples were stored at 4°C immediately after collection and analyzed within 24 hours of phlebotomy. Before analysis, samples were equilibrated to room temperature and gently inverted 8–10 times to ensure homogeneous cell suspension.

During measurement, the analyzer automatically maintained the sample chamber at 37 ± 0.1°C to minimize temperature-dependent variation in blood viscosity.

The analyzer was calibrated monthly using manufacturer-provided standard reference viscosity fluids. Daily internal quality-control materials were analyzed before routine testing. The between-day coefficients of variation were 2.90% for systolic blood viscosity (SBV) and 3.24% for diastolic blood viscosity (DBV).

Reference intervals were established in accordance with the Clinical and Laboratory Standards Institute (CLSI) EP28-A3c guideline. The reference intervals were 2.99–4.86 mPa·s for SBV and 6.21–11.45 mPa·s for DBV in men, and 2.68–4.43 mPa·s for SBV and 5.36–10.96 mPa·s for DBV in women.

All measurements were performed in accordance with standardized laboratory procedures.

**Laboratory workflow and measurement feasibility**

The study was conducted at the Kangbuk Samsung Hospital Total Healthcare Center, one of the largest health screening centers in Korea, which performs approximately 300,000 health examinations annually. In South Korea, employers are legally required to provide periodic health examinations under the Occupational Safety and Health Act, resulting in a large volume of routine health screening each year. Kangbuk Samsung Hospital provides health examinations for employees of several large corporate groups, contributing to one of the highest annual health-screening volumes in the country.

Although whole blood viscosity (WBV) measurement is an optional examination, the study period (January–April 2021) coincided with a promotional event, resulting in substantially increased uptake of WBV testing. During the study period, the laboratory operated **8 hours per day, 6 days per week**, was equipped with **two ZL6000P cone-plate blood rheology analyzers**, and required an average of **approximately 1.5 minutes per sample** for WBV measurement.

**Supplementary Table 1. Baseline characteristics of the overall study population**

| Sex | Total | Male | Female | P value |
| --- | --- | --- | --- | --- |
| n | 38,574 | 21,914 | 16,660 |  |
| Age, years | 40.81±8.79 | 40.99±8.54 | 40.58±9.11 | <0.001 |
| BMI, kg/m^2^ | 23.76±3.54 | 25.05±3.09 | 22.05±3.36 | <0.001 |
| Metabolic Syndrome, n | 4,524 | 3,619 | 905 | <0.001 |
| Waist circumference, cm | 82.43±10.26 | 87.42±8.29 | 75.79±8.75 | <0.001 |
| Systolic BP, mmHg | 109.05±11.54 | 112.45±10.25 | 104.58±11.60 | <0.001 |
| Diastolic BP, mmHg | 69.36±8.17 | 71.46±7.82 | 66.59±7.78 | <0.001 |
| Total cholesterol, mg/dL | 194.68±33.76 | 196.59±34.03 | 192.15±33.24 | <0.001 |
| HDL-c, mg/dL | 61.20±16.21 | 54.52±12.89 | 69.99±15.94 | <0.001 |
| LDL cl, mg/dL | 127.33±32.31 | 132.78±32.11 | 120.17±31.15 | <0.001 |
| Triglyceride, mg/dL | 114.36±76.66 | 133.95±85.12 | 88.59±53.92 | <0.001 |
| Fasting Glucose, mg/dL | 95.69±12.93 | 97.64±13.20 | 93.14±12.10 | <0.001 |
| HbA1c, % | 5.44±0.48 | 5.50±0.49 | 5.36±0.44 | <0.001 |
| AST, IU/L | 23.24±17.16 | 25.70±17.86 | 20.02±15.62 | <0.001 |
| ALT, IU/L | 25.18±22.15 | 31.85±23.88 | 16.41±15.83 | <0.001 |
| GGT, IU/L | 29.46±32.58 | 38.26±37.92 | 17.89±18.20 | <0.001 |
| Protein, g/dL | 7.44±0.39 | 7.46±0.38 | 7.41±0.41 | <0.001 |
| Albumin, g/dL | 4.81±0.26 | 4.88±0.25 | 4.72±0.26 | <0.001 |
| WBC, x10^3^/uL | 5.71±1.49 | 5.78±1.43 | 5.62±1.55 | <0.001 |
| RBC, x10^3^/uL | 4.82±0.44 | 5.08±0.32 | 4.47±0.30 | <0.001 |
| Hb, g/dL | 14.63±1.44 | 15.56±0.89 | 13.41±1.06 | <0.001 |
| Hct, % | 43.16±3.69 | 45.47±2.40 | 40.12±2.78 | <0.001 |
| SBV, mPa·s | 3.84±0.50 | 4.07±0.45 | 3.53±0.37 | <0.001 |
| DBV, mPa·s | 9.79±1.67 | 10.40±1.74 | 8.99±1.16 | <0.001 |
| Diabetes mellitus, n (%) | 1332 (3.45%) | 956 (4.36%) | 376 (2.26%) | <0.001 |
| Hypertension, n (%) | 3712 (9.62%) | 2860 (13.05%) | 852 (5.11%) | <0.001 |
| Dyslipidemia, n (%) | 9186 (23.81%) | 6756 (30.83%) | 2430 (14.59%) | <0.001 |
| Alcohol consumption, n (%) | 3482 (9.03%) | 3271 (14.93%) | 211 (1.27%) | <0.001 |
| Smoking, n (%) | 17113 (52.14%) | 11427 (56.99%) | 5686 (44.52%) | <0.001 |

Data are presented as mean ± standard deviation (SD) or number (%). Continuous variables were compared using independent-samples t-tests, and categorical variables were compared using the χ² test. BMI, body mass index; BP, blood pressure; HDL-c, high-density lipoprotein cholesterol; LDL-c, low-density lipoprotein cholesterol, AST, aspartate aminotransferase; ALT, alanine aminotransferase, GGT, gamma-glutamyl transferase; WBC, white blood cell count; RBC, red blood cell count; Hb, Hemoglobin; Hct, hematocrit; SBV, systolic blood viscosity; DBV, diastolic blood viscosity

**Supplementary Table 2. Derivation of the weighted MetS score and sensitivity analysis of its association with blood viscosity**

**Part A. Component weights derived from a crude logistic regression model of CVD**

| **MetS component** | **β coefficient (weight)** | **Standard Error** | **P value** |
| --- | --- | --- | --- |
| Triglyceride ≥150 mg/dL | −0.042 | 0.143 | 0.770 |
| HDL-cholesterol <40/50 mg/dL | 0.166 | 0.182 | 0.361 |
| Hypertension | 1.214 | 0.128 | <0.001 |
| Fasting glucose ≥100 mg/dL | 0.386 | 0.125 | 0.002 |
| Waist circumference ≥90/85 cm | 0.138 | 0.130 | 0.287 |

**Part B. Sensitivity analysis using the weighted MetS score**

| Outcome | β coefficient | 95% CI | P value |
| --- | --- | --- | --- |
| SBV | 0.127 | 0.119-0.135 | <0.001 |
| DBV | 0.283 | 0.254-0.311 | <0.001 |

Component weights (Part A) were derived from a crude logistic regression model using cardiovascular disease (angina, myocardial infarction, stroke, or cerebral hemorrhage/infarction), as recorded in the health-screening dataset, as the outcome and the five MetS components as predictors within the same cross-sectional cohort. These coefficients were used to construct the weighted MetS score for the sensitivity analysis presented in Part B: weighted score = (−0.042 × TG criterion) + (0.166 × HDL criterion) + (1.214 × Hypertension) + (0.386 × Fasting glucose) + (0.138 × Waist circumference). The weighted MetS score was then entered as the independent variable in crude linear regression models with SBV and DBV as the dependent variables. CVD, cardiovascular disease; MetS, metabolic syndrome; TG, triglyceride; HDL, high-density lipoprotein; SBV, systolic blood viscosity; DBV, diastolic blood viscosity; CI, confidence interval; TG, triglyceride; HDL, high-density lipoprotein.

**Supplementary Table 3. Independent contributions of individual metabolic syndrome components to systolic and diastolic blood viscosity**

| **Outcome** | **MetS component** | **β coefficient (95% CI) P value** | |
| --- | --- | --- | --- |
| SBV | Triglyceride ≥150 mg/dL | 0.237(0.224 - 0.250 | <0.001 |
|  | HDL-cholesterol <40/50 mg/dL | -0.040(-0.057 - -0.023) | <0.001 |
|  | Hypertension | 0.081(0.066 - 0.095) | <0.001 |
|  | Fasting glucose ≥100 mg/dL | 0.074(0.063 - 0.085) | <0.001 |
|  | Waist circumference ≥90/85 cm | 0.167(0.155 - 0.179) | <0.001 |
| DBV | Triglyceride ≥150 mg/dL | 0.568(0.524 - 0.611) | <0.001 |
|  | HDL-cholesterol <40/50 mg/dL | -0.197(-0.256 - -0.137) | <0.001 |
|  | Hypertension | 0.169(0.119 - 0.219) | <0.001 |
|  | Fasting glucose ≥100 mg/dL | 0.153(0.114 - 0.192) | <0.001 |
|  | Waist circumference ≥90/85 cm | 0.419(0.379 - 0.459) | <0.001 |

β coefficients (95% CIs) were estimated using multivariable linear regression models with systolic blood viscosity (SBV) or diastolic blood viscosity (DBV) as the dependent variable and all five metabolic syndrome (MetS) components entered simultaneously as independent variables. **SBV**, systolic blood viscosity; **DBV**, diastolic blood viscosity; **MetS**, metabolic syndrome; Tg, triglyceride; **HDL**, high-density lipoprotein; CI, confidence interval.

**Supplementary Table 4. Sex-by-viscosity interaction analyses for metabolic syndrome**

| **Interaction term** | **OR (95% CI) Model 1 (Unadjusted)** | **OR (95% CI) Model 2 (Hct-adjusted)** | **OR (95% CI)**  **Model 3 (Fully adjusted)** | **P value** |
| --- | --- | --- | --- | --- |
| SBV × sex | 1.321 (1.255–1.425) | 1.488 (1.248–1.774) | 1.166 (1.058-1.284) | <0.001 |
| DBV × sex | 1.362 (1.241–1.494) | 1.126 (1.053–1.202) | 1.211 (1.077-1.357) | <0.001 |

Odds ratios (ORs) were estimated using sex-by-viscosity interaction terms in logistic regression models. Model 1: unadjusted; Model 2: adjusted for hematocrit; Model 3: adjusted for hematocrit, age, smoking status, estimated glomerular filtration rate (eGFR), white blood cell count (WBC), AST, and ALT. ORs represent the interaction between sex and a 1-SD increase in SBV or DBV. SBV, systolic blood viscosity; DBV, diastolic blood viscosity; OR, odds ratio; CI, confidence interval; Hct, hematocrit; eGFR, estimated glomerular filtration rate; WBC, white blood cell count.

**Supplementary Table 5. Difference in blood viscosity between participants with and without metabolic syndrome**

|  | **MetS(-)** | **MetS(+)** | **MetS(-), Women** | **MetS(-), Men** | **MetS(+), Women** | **MetS(+), Men** |
| --- | --- | --- | --- | --- | --- | --- |
| SBV (mPa·s , mean±SE) | 3.824 ± 0.002 | 3.947 ± 0.005 | 3.796 ± 0.003^a^ | 3.847 ± 0.003^b^ | 3.939 ± 0.012^c^ | 3.958 ± 0.006^c^ |
| 95% CI | 3.821-3.828 | 3.937-3.957 | 3.789-3.802 | 3.841-3.853 | 3.916-3.962 | 3.946-3.97 |
| DBV (mPa·s , mean±SE) | 9.772 ± 0.007 | 9.954 ± 0.021 | 9.747 ± 0.014^a^ | 9.792 ± 0.012^a^ | 9.964 ± 0.046^b^ | 9.959 ± 0.024^b^ |
| 95% CI | 9.758-9.787 | 9.913-9.994 | 9.721-9.774 | 9.769-9.815 | 9.875-10.054 | 9.912-10.005 |

Adjusted means were estimated using analysis of covariance (ANCOVA) with sex and hematocrit as covariates. Overall group differences were significant (p < 0.001). Post-hoc pairwise comparisons were performed, and groups sharing the same superscript letter were not significantly different. SBV, systolic blood viscosity; DBV, diastolic blood viscosity; MetS, metabolic syndrome; SE, standard error; CI, confidence interval; ANCOVA, analysis of covariance.

| Marker | AUC | 95% CI | Sensitivity | Specificity |
| --- | --- | --- | --- | --- |
| Total SBV | 0.687 | 0.680-0.695 | 0.735 | 0.546 |
| Total DBV | 0.662 | 0.654-0.670 | 0.824 | 0.445 |
| Men SBV | 0.594 | 0.584-0.604 | 0.599 | 0.539 |
| Men DBV | 0.549 | 0.539-0.560 | 0.463 | 0.615 |
| Women SBV | 0.659 | 0.640-0.677 | 0.581 | 0.650 |
| Women DBV | 0.589 | 0.569-0.609 | 0.382 | 0.750 |

**Supplementary Table 6. Diagnostic Performance of WBV for Metabolic Syndrome**

Diagnostic performance was assessed using receiver operating characteristic (ROC) curve analysis. SBV, systolic blood viscosity; DBV, diastolic blood viscosity; AUC, area under the receiver operating characteristic curve; CI, confidence interval.

## Supplementary Table 7. Comparative reclassification analyses

| Comparison  (vs. SBV as reference) | NRI (95% CI) | P value | IDI (95% CI) | P value |
| --- | --- | --- | --- | --- |
| DBV | -0.454 (-0.528 to -0.370) | <0.001 | -0.013 (-0.017 to -0.010) | <0.001 |
| Tg/HDL-C | 1.210 (1.176–1.238) | <0.001 | 0.258 (0.242–0.273) | <0.001 |

Comparative model performance was evaluated using net reclassification improvement (NRI) and integrated discrimination improvement (IDI) analyses, with SBV as the reference model. Positive NRI and IDI values indicate improved reclassification and discrimination compared with SBV. SBV, systolic blood viscosity; DBV, diastolic blood viscosity; TG/HDL-C, triglyceride-to-high-density lipoprotein cholesterol ratio; NRI, net reclassification improvement; IDI, integrated discrimination improvement.

**Supplementary Figure 2. Regression tree analysis identifying key metabolic determinants of blood viscosity.**

**SBV**

**DBV**

Regression trees were generated using recursive partitioning (rpart package in R) and are shown separately for systolic blood viscosity (SBV, upper panel) and diastolic blood viscosity (DBV, lower panel). Each node displays the mean ± standard error (SE) and sample size. Sex (female = 1) was specified as the first branching variable, followed by binary indicators of individual MetS components: tgC (triglyceride criterion), wcC (waist circumference criterion), and glucC (fasting glucose criterion), each coded as 0 (criterion not met) or 1 (criterion met). SBV, systolic blood viscosity; DBV, diastolic blood viscosity; MetS, metabolic syndrome; Tg, triglyceride; WC, waist circumference.
